# The Engineering Side of Hospital Work

**Published:** 1905-04-22

**Authors:** 


### HOSPITAL ADMINISTRATION.

### CONSTRUCTION AND ECONOMICS.

### THE ENGINEERING SIDE OF HOSPITAL WORK.

IX.—LAUNDRIES. DRYING THE CLOTHES.

(*Continued from page* 58.)

Heating and Circulating the air in the Drying Closets.

The arrangements for heating and circulating the air in the closets are various, but practically they are the same as those for creating draught for steam boilers, and for heating the air passing to the boiler furnaces, with certain modifications. The draught may be arranged by means of a chimney, the so-called Natural Draught, or by fans; and the fans may operate to force the air into the closets, or to suck it through them. Where the air is forced through the closet the arrangement is termed Forced Draught, and where it is sucked through it is called Induced Draught. All three terms are bad. In all three cases the cause which determines the passage of the air through the closets is the creation of a difference of pressure in the air between two sides of the closet, or say the top and bottom of the left hand side and the right hand side. With the chimney the difference of pressure is caused by the lesser weight of the column of hot air in the chimney, as against the weight of an equivalent column of atmospheric air, the hot air passing from the closets to the chimney, and the weight of the column of air outside forcing air over the heating apparatus, through the closets, and up the chimney. With Forced Draught the fan, which is in the path of the air before it reaches the chamber, creates a higher pressure in front than exists at the back of the chamber, this being assisted by the expansion of the air in the process of heating. With Induced Draught the fan, which is placed behind the chamber, in the course of the air, creates a lower pressure behind or beyond the chamber than in front. In either case the difference of pressure, continuously maintained, keeps up a continual stream of air through the chamber. Heating the air is accomplished by placing either steam coils, or flues from coal furnaces, in the path of the air. The arrangements vary with each designer and each maker, but all work on certain main lines. Steam-pipes formed into grids or coils are placed in a convenient position, and the air is led over them by ducts, the air passing on to the closet. The pipes may be above or below or at the side of the chamber, according to the fancy of the engineer, and the air may be heated before it reaches the chamber, or in the chamber, again according to the fancy of the engineer. For large plants, where there is plenty of steam available, steam-heating is the most convenient Steam forms, as already explained, a very convenient agent for distributing heat. With small plants, the drying closets are sometimes built round a furnace, the gases of which are carried through a flue arranged in the path of the air that is to be heated, the result is the same. With steam the heat is obtained at second-hand, with a coal furnace at first hand; but it does not follow that the furnace is the most economical. Usually steam is the most economical, because there is, or should be, less waste of heat in large furnaces, such as would rule with a battery of boilers, than in small isolated furnaces, and though there is some loss in distributing steam, this should not make up the difference, if proper precautions are taken to thermally insulate the pipes, etc.

Drying-rooms.

Drying-rooms are arranged on very much the same lines as drying-closets. They occupy a larger space than drying-closets, and the air passing through them for the purpose of drying the clothes is not always heated, except in so far as may be by taking it from rooms, such as those used for ironing, that are at a comparatively high temperature. The question of the relative efficiency of drying-rooms, and drying-closets will depend upon the conditions, such as the space available, and the general arrangements. Other things being the same drying-rooms would not be so efficient as drying closets, unless special arrangements were made to thermally insulate the rooms. With the drying-closets, though the heated chambers are not insulated thermally, beyond such insulation as good stout brick walls will confer, the whole arrangement is so confined that a comparatively small proportion of the heat delivered by the steam-pipes, or by the flues, will be carried away, except by the warmed air, and hot-flue gases themselves. The air and the gases also are obliged to deliver the largest possible percentage of the heat they carry under the drying-closet arrangements, while with the drying-room there will be sure to be considerable sources of leakage. On the other hand, with drying-rooms the cost of heating the air may be saved, and the total cost of drying for this part of the work will be the cost of running the fans which keep up the air circulation. Air leakage will affect this item very considerably. If only 60 per cent. of the air forced through the drying-room is actually available for absorbing moisture from the clothes, the cost of running the fan will be approximately doubled. The fact that the heated air rapidly increases its ability to absorb moisture also must not be lost sight of in this matter. For practical purposes there is only one method that is reliable in all these cases, and that is the balance-sheet. Put on one side the total cost of drying a certain quantity of clothes by drying-rooms, and on the other side the cost for the same number by drying-closets, and compare. But in drawing the balance-sheet, be very careful that you get all the items into the account. It is the easiest matter in the world to draw a most flourishing balance-sheet if you leave out plenty of the items on one side. Interest on plant and repairs, which often form a most important item, should not be left out, also the labour on both sides, and where the item has a value, as in London, the rent of the ground occupied or the interest on the sum expended in its purchase. It will be understood that the air which has done its work in drying the clothes in the closets, is carried off by ducts or flues. In some cases it is passed through another room on its way to the atmosphere. In the next article, the writer will deal with finishing apparatus, and the apparatus he found in use in the hospitals he visited.

